# improving Pain mAnagement for childreN and young people attendeD by Ambulance (PANDA): protocol for a realist review.

**DOI:** 10.3310/nihropenres.13627.3

**Published:** 2025-01-30

**Authors:** Georgie Nicholls, Georgette Eaton, Marishona Ortega, Kacper Sumera, Michael Baliousis, Jessica Hodgson, Despina Laparidou, Aloysius Niroshan Siriwardena, Paul Leighton, Sarah Redsell, Bill Lord, Tatiana Bujor, Gregory Adam Whitley

**Affiliations:** 1 Community and Health Research Unit, University of Lincoln, Lincoln, England, LN6 7FS, UK; 2 London Ambulance Service NHS Trust, London, England, SE1 8SD, UK; 3 Nuffield Department of Primary Care Health Sciences, University of Oxford, Oxford, England, OX2 6GG, UK; 4 Libraries and Learning Skills, University of Lincoln, Lincoln, England, LN6 7TS, UK; 5 East Midlands Ambulance Service NHS Trust, Nottingham, England, NG8 6PY, UK; 6 School of Psychology, University of Lincoln, Lincoln, England, LN5 7TS, UK; 7 Nottingham University Hospitals NHS Trust, Nottingham, England, NG5 1PB, UK; 8 School of Medicine, University of Nottingham, Nottingham, England, NG7 2RD, UK; 9 Applied Health Research Building (Building 42), University of Nottingham, Nottingham, England, NG7 2RD, UK; 10 Faculty of Medicine & Health Sciences, University of Nottingham, Nottingham, England, NG7 2UH, UK; 11 Monash University, Clayton, Victoria, Australia; 12 The Medical School, Newcastle University, Newcastle upon Tyne, England, NE2 4HH, UK; 13 Clinical Audit and Research Unit, East Midlands Ambulance Service NHS Trust, Lincoln, England, LN4 2HL, UK

**Keywords:** Acute Pain, Analgesia, Child, Emergency Medical Services, Paramedics, Paediatrics

## Abstract

**Background:**

Each year in England, 450,000 children and young people (CYP) under 18 years of age are transported by ambulance to emergency departments. Approximately 20% of these suffer acute pain caused by illness or injury. Pain is a highly complex sensory and emotional experience. The intersection between acute pain, unwell CYP and the unpredictable pre-hospital environment is convoluted. Studies have shown that prehospital pain management in CYP is poor, with 61% of those suffering acute pain not achieving effective pain relief (abolition or reduction of pain score by 2 or more out of 10) when attended by ambulance. Consequences of poor acute pain management include altered pain perception, post-traumatic stress disorder and the development of chronic pain. This realist review will aim to understand how ambulance clinicians can provide improved prehospital acute pain management for CYP.

**Methods:**

A realist review will be conducted in accordance with the Realist And Meta-narrative Evidence Syntheses: Evolving Standards (RAMESES) guidance. A five-stage approach will be adopted; 1) Developing an Initial Programme Theory (IPT): develop an IPT with key stakeholder input and evidence from informal searching; 2) Searching and screening: conduct a thorough search of relevant research databases and other literature sources and perform screening in duplicate; 3) Relevance and rigour assessment: assess documents for relevance and rigour in duplicate; 4) Extracting and organising data: code relevant data into conceptual “buckets” using qualitative data analysis software; and 5) Synthesis and Programme Theory (PT) refinement: utilise a realist logic of analysis to generate context-mechanism-outcome configurations (CMOCs) within and across conceptual “buckets”, test and refine the IPT into a realist PT.

**Conclusion:**

The realist PT will enhance our understanding of what works best to improve acute prehospital pain management in CYP, which will then be tested and refined within a realist evaluation.

**Registration:**

PROSPERO Registration: CRD42024505978

## Plain language summary

Each year in England approximately 90,000 children and young people under 18 years of age suffer with acute pain and require transport by ambulance to emergency departments. The pain may have been caused by injuries such as wounds, burns or broken bones, or by illnesses such as tummy pain. Paramedics and other ambulance clinicians aim to reduce pain at the scene and during hospital transport. Whilst access to pain management is considered a fundamental human right, around 60% of children and young people who require an ambulance do not have their acute pain treated effectively. Without effective pain treatment, adverse consequences such as post-traumatic stress disorder may occur. We aim to understand how ambulance clinicians can provide improved prehospital acute pain management for children and young people.

We will develop a theory about what is most important when considering the improvement of acute pain management for children and young people attended by ambulance. We will use published evidence, opinions from experts in the field, such as paramedics, paramedic educators and clinical leaders, and opinions from members of a Young Persons Advisory Group, to help us create this theory. We will then conduct a thorough search for any published documents that can help us test this theory. Such documents may include published journal articles, clinical practice guidelines, dissertations or newspaper articles for example. We will then use the information within all the relevant documents to test our theory and make refinements. This will allow us to produce a refined theory of what works best to improve acute pain suffered by children and young people who need an ambulance. We will then test and refine this theory in a future study by asking children, young people, parents, carers and ambulance clinicians about parts of the theory.

## Introduction

Ambulance services across England transport 4.7 million patients to emergency departments (ED) each year, of which 450,000 (9.5%) are children and young people (CYP) under 18 years of age ^1^. Acute pain caused by injury or illness is a common symptom presented to ambulance clinicians, and is suffered by approximately 20% of transported CYP ^2^. Paramedics and other ambulance clinicians aim to reduce pain at the scene and during hospital transport ^3^, however this can be challenging in CYP due to a variety of barriers. These include fear and anxiety – which can distort pain assessment ^4^, environmental factors, staff feeling ill prepared to manage pain due to limited education and training and low exposure rates to CYP, and difficulties in assessing and treating pain, particularly regarding limited analgesic options and difficulties of analgesic administration in CYP ^5^.

Access to pain management is considered a fundamental human right ^6^ and effective pain relief has been identified as a key quality outcome measure for ambulance services ^7^. Despite this, prehospital pain management in CYP is considered poor ^8, 9^. Ambulances, equipment and staff uniform, are often not tailored towards CYP, and as such, an ambulance call out can be a frightening experience ^5^. A recent study found that only 39% of CYP who suffered acute pain in the prehospital setting achieved effective pain relief (defined as the abolition or reduction of pain >=2 points on a 10-point scale) ^2, 10– 12^. The consequences of poor acute pain management may include the development of post-traumatic stress disorder ^13, 14^, altered pain perception ^15, 16^, and the subsequent development of chronic pain ^17, 18^.

Typically, within the UK CYP who require an emergency ambulance for acute pain are attended by a registered paramedic or emergency medical technician (EMT). In more critical cases, they may also be attended by paramedics with enhanced skills or doctors via a specialist service. Pain is typically measured using the Face, Legs, Activity, Cry, Consolability (FLACC) scale for those unable to communicate their pain, the Wong and Baker FACES® scale for those aged 3 years and above, and the numeric pain rating scale for those aged around 8 years and above ^3^.

The scope of practice between these clinicians vary significantly, with EMTs able to administer only nitrous oxide and simple oral analgesics such as paracetamol and ibuprofen, to paramedics who can also administer paracetamol (intravenous) along with morphine sulfate (oral, subcutaneous, intramuscular, intravenous) ^19^, to paramedics with enhanced skills who can also administer intravenous ketamine. Doctors have a much greater scope of analgesic options available, including fentanyl and diamorphine. The limited range of analgesic options available to UK ambulance clinicians are in part due to legal restrictions ^19, 20^ which preclude the use of key controlled drugs such as fentanyl by UK registered paramedics, which can be administered intranasally ^21^ or via a lozenge ^22^. Nitrous oxide is widely available to UK ambulance clinicians but is challenging to administer to CYP due to its cumbersome nature ^23^. Methoxyflurane offers a promising alternative due to its light weight and ease of use, but is not currently licenced for children in the UK ^24^, with results of a major clinical trial due soon ^25^. These legal restrictions may change in the wake of the Manchester Arena Enquiry ^26^ and the recent call to arms to “make children’s pain matter” by Eccleston *et al.* ^27^, however reliance for improvement should not rest on single strategies. Whilst an increased range of analgesics would be welcome, it would unlikely resolve the complex challenge of providing effective prehospital pain management for CYP ^28^, therefore other strategies should be explored.

This realist review will aim to understand how ambulance clinicians can provide improved prehospital acute pain management for CYP. The review will focus on potential behaviour change intervention components that could be aimed at ambulance clinicians.

## Methods

### Patient and public involvement

A Young Persons Advisory Group (YPAG) has been set up to advise on the initial design of the PANDA Study ^29^. The YPAG group will continue to provide input within this realist review. The group was recruited from a state funded secondary school and comprises 25 members in total. The age range of members spans from 12 to 18 years, 60% are female (n = 15), 64% White (n = 16), 24% Asian or Asian British (n = 6) and 13% Other or Mixed ethnicity (n = 3). Four of the YPAG members have experience of being in an ambulance with a painful condition, three have been in an ambulance for other reasons and four have witnessed friends or family members going into an ambulance. The YPAG group will meet to provide insights and suggestions to develop and refine the initial programme theory (IPT) and to assist in the interpretation of the synthesis and refinement of the realist programme theory.

An established patient and public involvement group based at the University of Lincoln (the Healthier Ageing Patient and Public Involvement (HAPPI) group)), was involved during the initial design of the PANDA Study. The HAPPI group will continue to be involved at key stages throughout this realist review, particularly to assist with the interpretation of findings. The HAPPI Group members will provide input from a “public” perspective and will also bring external expertise to the project from their links to other patient and public involvement groups and from experience of advising several other prehospital ambulance-based research projects.

### Study design

The overall PANDA Study is a realist informed complex intervention development and feasibility study, consisting of a realist review, a realist evaluation, consensus workshops and a feasibility trial ^30^. The aim of the PANDA Study is to develop and test an intervention to improve pre-hospital pain management for CYP by exploring what interventions work, for whom, in what context and how. This paper reports the protocol for the realist review component of the PANDA Study.

The PANDA Study will be framed within a realist approach as described by Pawson ^31– 33^, which aligns to the Medical Research Council guidelines for complex intervention development ^34^. A realist approach seeks to understand why, how, to what extent, for whom and in what circumstances a programme or intervention works ^35^. It assumes that interventions or programmes themselves do not *cause* outcomes, rather, it is the resources offered by the intervention that trigger a response from the participant through underlying unseen **mechanisms**, that cause **outcomes**, within a specific **context** ^35^. These context-mechanism-outcome configurations (CMOCs) are the foundation on which programme theory is built and may be informed by primary (realist evaluation) or secondary (realist review) data ^32, 33^.

A realist review will be conducted, following the Realist And Meta-narrative Evidence Syntheses: Evolving Standards (RAMESES) guidance ^35^. The realist review has been registered on PROSPERO, the international prospective register of systematic reviews (CRD42024505978) ^36^. There are currently no equator network publication standards for realist review protocols, therefore we used the realist review publication standards as a framework to report this protocol ^37^

The objectives for this realist review are:

1. To develop an IPT to map the key processes of prehospital acute pain management in CYP.
2. To focus on specific areas of the IPT to explore potential behaviour change intervention components aimed at ambulance clinicians and determine; for whom they work; in what circumstances; how; and to what extent they work.
3. To refine the IPT into a realist programme theory supported by context, mechanism, outcome configurations (CMOCs).

We will adopt a five-stage approach to conduct this realist review, informed by the RAMSES guidance ^35^, Pawson ^32^ and a recent realist review ^38^. These steps will include:

1. Locating existing theories and developing IPT
2. Searching and screening
3. Relevance and rigour assessment
4. Extracting and organising data
5. Synthesis and Programme Theory (PT) refinement

## Step 1: Locating existing theories and developing IPT

The first stage of this realist review will involve locating existing theories about prehospital pain management in CYP. This will involve informal searches to identify key theories in the field, what the predictors, barriers and facilitators are for effective prehospital pain management in CYP, and which components of the process are considered most important. Whilst this process is subjective, we will offset this by involving multiple key stakeholder groups in the development of the IPT, namely an Ambulance Clinician Advisory Group (ACAG), a Young Persons Advisory Group (YPAG) and a PANDA Study Realist Review Working Group. The inclusion of these groups is important as realist reviews are driven by stakeholders, enabling the inclusion of multiple perspectives ^39, 40^.

Informal searches will be conducted to ensure that relevant documents are identified, and key data are incorporated within the IPT development process. One member of the review team (GAW) has access to a large number of relevant documents (including journal articles, theses and book chapters), having recently completed a PhD on the topic of prehospital pain management in children. Combining these documents with stakeholder input and iterative discussion within the PANDA Study Realist Review Working Group will enable the development of our IPT.

## Step 2: Searching and screening

With the assistance of an academic librarian (MO), the IPT will be used as a framework to develop a comprehensive search strategy.

**Database Search:** Relevant keywords, subject headings and Boolean operators will be used to search major bibliographic databases. The EBSCOHost platform will be used to search MEDLINE, Cumulative Index to Nursing & Allied Health (CINAHL) Complete, PsycINFO, Web of Science Core Collection, Education Source, Education Resources Information Centre (ERIC), and The Cochrane Library

**Searching Other Resources:** The clinical trials registry ISRCTN will be searched, along with other literature sources such as ProQuest, including ProQuest Dissertations and Theses, and Google. Expert knowledge will also be used to identify relevant documents not found during these searches. Forward and backward citation tracking will be conducted for all included documents.

Additional searches may be required during the realist review as programme theory testing and refinement progresses. Informed by a recent systematic mixed studies review on this topic that searched core databases from inception, and included papers from 2006 to 2020 ^5^, we will restrict our search to documents published from January 2000 onwards, due to known limited evidence prior to 2000 ^5^.

### Inclusion criteria

In-line with a realist philosophy of science ^32^, all sources of information may contribute to the development of realist programme theory, therefore we will include, where relevant, research articles, clinical practice guidelines, policy documents, websites of professional bodies or reputable organisations, conference abstracts, theses and dissertations, along with curricula.

In addition to the wide range of documents eligible for inclusion, only documents reported in English (due to time and resource constraints) and involving or aimed at the following populations will be included:

- Ambulance clinicians (including but not limited to paramedics, emergency medical technicians, prehospital emergency nurses) who attend to CYP suffering acute pain.
- CYP suffering acute pain in the prehospital setting.
- Parents/carers of CYP suffering acute pain in the prehospital setting.

### Exclusion criteria

Documents will be excluded if they are:

- Based in the battlefield, in-hospital, primary care or helicopter emergency medical service (HEMS) setting. Documents from these settings would not be representative of standard ambulance service practice.
- Focussed on chronic pain.

Documents reporting prehospital acute pain management data for children, young people *and* adults will be excluded if the data for CYP under 18 years of age cannot be isolated and extracted.

Identified documents from the database search will be imported to Covidence (copyright licence obtained) software and screened independently in duplicate. Documents will be screened first by title and abstract, followed by full text review against the inclusion and exclusion criteria. Documents identified from other resources will be added to and managed within MS Excel software and screened independently in duplicate. These documents will be subject to an initial screen, similar in nature to the title and abstract screen, followed by a full-text screen, where the full document will be reviewed against the inclusion and exclusion criteria. Disagreements on inclusion will be resolved through discussion, or involvement of a third member of the review team.

## Step 3: Relevance and rigour assessment

Documents deemed to meet the inclusion criteria will then be assessed for relevance and rigour, as per the RAMESES guidelines ^35^. Relevance relates to the ability of data within a document to contribute to the testing and refinement of programme theory, and rigour relates to whether the methods used to generate the relevant data are credible and trustworthy ^35^. Whilst there is relative consensus regarding the methods to assess the relevance of documents within a realist review, there is substantial uncertainty among academics regarding how best to assess rigour ^41^. Given the adoption of a realist philosophy of science ^32^, using a checklist approach to quality assessment, as standard within a systematic review ^42^, is less helpful in a realist review due to the inclusive nature of data from a wide variety of sources. We will therefore not use critical appraisal/quality checklists as part of our rigour assessment.

### Relevance

The assessment of relevance will be dichotomous (yes/no) and conducted within MS Excel software. Two reviewers will separately assess relevance of a small sample of documents and discuss with the PANDA Study Realist Review Working Group as a benchmarking exercise. If agreement is achieved, the remaining documents will be assessed for relevance, in duplicate. Disagreements will be resolved through discussion, or involvement of a third member of the review team. A third reviewer will assess 10% of reviewed documents to ensure consistency. Documents deemed not relevant will be excluded from the review.

### Rigour

Rigour will be assessed in duplicate by two reviewers independently and based on reviewer judgement of document trustworthiness. A rating scale will be used to determine the rigour of each document (low, moderate or high rigour). Disagreements will be settled through discussion, or the involvement of a third reviewer. A third reviewer will assess 10% of reviewed documents to ensure consistency.

Rigour will be assessed at the level of the data and at the level of the programme theory ^41^. Documents deemed highly trustworthy and credible at the level of the data (for example where clear methods of data production are described and references for evidence sources are listed) and are coherent at the level of the programme theory and provide consilience and analogy (for example where the documents support the programme theory well), will be rated high.

Rigour will not be used as a reason for exclusion ^43^. Instead, CMOCs that are considered conceptually weak (i.e. the documents informing the CMOCs are mostly rated as low or moderate rigour) will be tested further through additional iterative searches, or within the realist evaluation.

The number of included documents will be reported using a PRISMA flow diagram ^42^.

## Step 4: Extracting and organising data

### Data extraction

Data extraction will occur in two phases.

1. The characteristics of included documents will be manually extracted into a Microsoft Word document, including bibliographic information and details about document type and population. This will form the summary of included documents table. This will be performed by one reviewer and verified in full by a second.
2. Included documents will then be uploaded to NVivo version 14 (copyright licence obtained) software for data extraction (coding). Data will not be extracted per se, rather the full documents will be uploaded for coding. Qualitative and quantitative text that is relevant to the IPT will be coded; this may consist of descriptions, findings or explanations of programmes or interventions that aim to improve prehospital acute pain management for CYP ^37^.

### Data organising

Coding will be deductive (based on the IPT), inductive (where new conceptual buckets arise) and retroductive (when inferring causal mechanisms within CMOC development). Text will be coded as “parent nodes” or “child nodes” iteratively and combined/expanded during the organising phase of analysis. Coding will be conducted by one reviewer, with 10% of coded documents checked by a second reviewer.

Codes assigned as “parent nodes” will be viewed as conceptual “buckets” ^44^. Text may be coded into more than one conceptual “bucket”.

## Step 5: Synthesis and programme theory refinement

### Synthesis

As more sections of coded text are added to each conceptual “bucket”, two reviewers will periodically pause to determine, as far as possible, what is functioning as context, mechanism and outcome, thereby creating CMOCs. This process will use a realist logic of analysis ^32^. This interpretation will be iterative in nature and reviewed extensively by the Working Group and key stakeholders to reduce researcher bias. Coded text from more than one conceptual “bucket” may be used to create CMOCs. For each developed CMOC, a new “parent node” will be created, with all the supporting data extracts for the CMOC added. This will ensure clean and clear traceability between source data, CMOC and programme theory.

Each developed and substantiated CMOC will contribute to the development and refinement of the realist programme theory. CMOCs that are unsubstantiated, either due to low rigour or conflicting data, may be tested further through additional iterative searches or through the realist evaluation.

Data to inform our interpretation of the relationships between contexts, mechanisms and outcomes will be sought across documents. There may be instances where data coded from documents contradict each other, or only supply part of the CMOC. We may juxtapose, reconcile, adjudicate, consolidate or situate (Pawson, 2006) findings throughout the analytic process, as necessary.

### Programme theory refinement

We will test the IPT with data collected and synthesised from this review and refine it into realist programme theory supported by CMOCs. As this review is not assessing a specific intervention or programme, but rather the process of prehospital acute pain management in CYP, we anticipate the realist programme theory to be segregated into stages based on outcome, progressing from proximal outcomes (focussed on ambulance clinicians – such as confidence and knowledge) to more distal outcomes (focussed on CYP – such as pain severity). The programme theory developed from a recent realist review will be used as a framework to develop our realist programme theory ^45^.

The realist programme theory we develop will then be used to develop and test a complex intervention aimed at ambulance clinicians in a future stage of the PANDA Study.

## Stakeholder involvement

In addition to the patient and public involvement groups, we will involve other key stakeholders including ambulance clinicians, academics, clinical and non-clinical psychologists.

### Ambulance clinician involvement

An Ambulance Clinician Advisory Group (ACAG) has been established, with expertise from the fields of clinical practice, education, and senior leadership, ranging from the South to the North of England, and Scotland. The group will meet at several stages of the review to provide insights and their expert knowledge to help with the development and refinement of the IPT, provide advice and feedback on the PT as it develops, identify any relevant literature that will assist with the research and to facilitate the interpretation of the findings.

### PANDA Study Realist Review Working Group

A bespoke PANDA Study Realist Review Working Group has been created for the realist review component of the PANDA Study, which consists of academics with expertise on realist methods, the prehospital setting and the population of CYP, clinicians, ambulance service representatives, along with clinical and non-clinical psychologists. The group will meet monthly, or more if required, to discuss the progress of the realist review and provide expertise at all stages of the review.

## Ethical considerations

Not required.

## Data Availability

No data are associated with this article.

## Software availability

NVivo 14 is a proprietary software, free alternatives such as QualCoder ( https://qualcoder.wordpress.com/) could be used. Covidence is a proprietary software, alternatives such as Rayyan ( https://www.rayyan.ai/) have a free membership option.

## References

[1] NHS Digital: Hospital accident & emergency activity 2021–22.2020; Accessed 19-Apr-2024. Reference Source

[2] Whitley GA Hemingway P Law GR : Predictors of effective management of acute pain in children within a UK ambulance service: a cross-sectional study. *Am J Emerg Med.* 2020;38(7):1424–1430. 10.1016/j.ajem.2019.11.043 31864872

[3] Joint Royal Colleges Ambulance Liaison Committee and Association of Ambulance Chief Executives: JRCALC Clinical Guidelines 2022.Bridgwater: Class Professional Publishing;2022. Reference Source

[4] Williams DM Rindal KE Cushman JT : Barriers to and enablers for prehospital analgesia for pediatric patients. *Prehosp Emerg Care.* 2012;16(4):519–526. 10.3109/10903127.2012.695436 22823931

[5] Whitley GA Hemingway P Law GR : The predictors, barriers and facilitators to effective management of acute pain in children by emergency medical services: a systematic mixed studies review. *J Child Health Care.* 2021;25(3):481–503. 10.1177/1367493520949427 32845710 PMC8422593

[6] Brennan F Lohman D Gwyther L : Access to pain management as a human right. *Am J Public Health.* 2019;109(1):61–65. 10.2105/AJPH.2018.304743 32941757 PMC6301399

[7] Turner J Siriwardena AN Coster J : Developing new ways of measuring the quality and impact of ambulance service care: the PhOEBE mixed-methods research programme. *Programme Grants Appl Res.* 2019;7(3). 10.3310/pgfar07030 31034193

[8] Samuel N Steiner IP Shavit I : Prehospital pain management of injured children: a systematic review of current evidence. *Am J Emerg Med.* 2015;33(3):451–454. 10.1016/j.ajem.2014.12.012 25572641

[9] Lord B Jennings PA Smith K : The epidemiology of pain in children treated by paramedics. *Emerg Med Australas.* 2016;28(3):319–324. 10.1111/1742-6723.12586 27147481

[10] Bulloch B Tenenbein M : Assessment of clinically significant changes in acute pain in children. *Acad Emerg Med.* 2002;9(3):199–202. 10.1111/j.1553-2712.2002.tb00244.x 11874775

[11] Bailey B Daoust R Doyon-Trottier E : Validation and properties of the verbal numeric scale in children with acute pain. *Pain.* 2010;149(2):216–221. 10.1016/j.pain.2009.12.008 20188471

[12] Tsze DS Hirschfeld G von Baeyer CL : Clinically significant differences in acute pain measured on self-report pain scales in children. *Acad Emerg Med.* 2015;22(4):415–22. 10.1111/acem.12620 25773461 PMC4390461

[13] Sheridan RL Stoddard FJ Kazis LE : Long-term posttraumatic stress symptoms vary inversely with early opiate dosing in children recovering from serious burns: effects durable at 4 years. *J Trauma Acute Care Surg.* 2014;76(3):828–832. 10.1097/TA.0b013e3182ab111c 24553556

[14] Saxe G Stoddard F Courtney D : Relationship between acute morphine and the course of PTSD in children with burns. *J Am Acad Child Adolesc Psychiatry.* 2001;40(8):915–921. 10.1097/00004583-200108000-00013 11501691

[15] Taddio A Katz J Ilersich AL : Effect of neonatal circumcision on pain response during subsequent routine vaccination. *Lancet.* 1997;349(9052):599–603. 10.1016/S0140-6736(96)10316-0 9057731

[16] Weisman SJ Bernstein B Schechter NL : Consequences of inadequate analgesia during painful procedures in children. *Arch Pediatr Adolesc Med.* 1998;152(2):147–149. 10.1001/archpedi.152.2.147 9491040

[17] Daoust R Paquet J Cournoyer A : Relationship between acute pain trajectories after an emergency department visit and chronic pain: a Canadian prospective cohort study. *BMJ Open.* 2020;10(12): e040390. 10.1136/bmjopen-2020-040390 33293313 PMC7722811

[18] McKillop HN Banez GA : A broad consideration of risk factors in pediatric chronic pain: where to go from here? *Children (Basel).* 2016;3(4):38. 10.3390/children3040038 27916884 PMC5184813

[19] The Human Medicines Regulations 2012.2012. 10.12968/bjcn.2012.17.9.445 23123490

[20] The Misuse of Drugs Regulations 2001.2001.

[21] Lord B Jennings PA Smith K : Effects of the introduction of intranasal fentanyl on reduction of pain severity score in children: an interrupted time-series analysis. *Pediatr Emerg Care.* 2019;35(11):749–754. 10.1097/PEC.0000000000001376 29200141

[22] Mahar PJ Rana JA Kennedy CS : A Randomized Clinical Trial of oral transmucosal fentanyl citrate versus intravenous morphine sulfate for initial control of pain in children with extremity injuries. *Pediatr Emerg Care.* 2007;23(8):544–548. 10.1097/PEC.0b013e318128f80b 17726413

[23] Whitley GA Hemingway P Law GR : Improving ambulance care for children suffering acute pain: a qualitative interview study. *BMC Emerg Med.* 2022;22(1): 96. 10.1186/s12873-022-00648-y 35659188 PMC9164349

[24] Electronic Medicines Compendium : PENTHROX 99.9%, 3 ml inhalation vapour, liquid.2023; Accessed 19 Apr-2024. Reference Source

[25] Hartshorn S Barrett MJ Lyttle MD : Inhaled methoxyflurane (Penthrox®) versus placebo for injury-associated analgesia in children-the MAGPIE trial (MEOF-002): study protocol for a randomised controlled trial. *Trials.* 2019;20(1): 393. 10.1186/s13063-019-3511-4 31272493 PMC6610896

[26] Saunders J : Manchester Arena Inquiry Volume 2: emergency response.2022; Accessed 06 Nov-2023. Reference Source

[27] Eccleston C Fisher E Howard RF : Delivering transformative action in paediatric pain: a *Lancet Child & Adolescent Health Commission*. *Lancet Child Adolesc Health.* 2021;5(1):47–87. 10.1016/S2352-4642(20)30277-7 33064998

[28] Lord B Jennings PA Smith K : Effects of the introduction of intranasal fentanyl on reduction of pain severity score in children: an interrupted time-series analysis. *Pediatr Emerg Care.* 2019;35(11):749–754. 10.1097/PEC.0000000000001376 29200141

[29] Whitley GA Siriwardena A Redsell S : 236 developing a Young Persons Advisory Group (YPAG) to inform the design of a study to improve pre-hospital pain management for Children and Young People (CYP). *BMJ Open.* 2022;12(Suppl 1):A2–A3. 10.1136/bmjopen-2022-EMS.5

[30] National Institute for Health and Care Research : Improving Pain mAnagement for childreN and young people attendeD by Ambulance (PANDA): a realist informed intervention development and feasibility study.2023; Accessed 23 Apr-2024. Reference Source

[31] Pawson R : The science of evaluation: a realist manifesto.SAGE Publications;2013. 10.4135/9781473913820

[32] Pawson R : Evidence-based policy: a realist perspective.SAGE Publications;2006. Reference Source

[33] Pawson R Tilley N : Realistic evaluation.SAGE Publications;1997. Reference Source

[34] Skivington K Matthews L Simpson SA : A new framework for developing and evaluating complex interventions: update of Medical Research Council guidance. *BMJ.* 2021;374: n2061. 10.1136/bmj.n2061 34593508 PMC8482308

[35] Wong G Greenhalgh T Westhorp G : Development of methodological guidance, publication standards and training materials for realist and meta-narrative reviews: the RAMESES (Realist And Meta-narrative Evidence Syntheses - Evolving Standards) project. *Health Serv Deliv Res.* 2014; 2(30). 10.3310/hsdr02300 25642521

[36] Horner G Siriwardena A Hodgson J : How can ambulance clinicians provide improved acute pain management for children and young people (PANDA Study)? A realist review.2024; Accessed 23 Apr-2024. Reference Source

[37] Wong G Greenhalgh T Westhorp G : RAMESES publication standards: realist syntheses. *BMC Med.* 2013;11(1): 21. 10.1186/1741-7015-11-21 23360677 PMC3558331

[38] Wong G Brennan N Mattick K : Interventions to improve antimicrobial prescribing of doctors in training: the IMPACT (IMProving Antimicrobial presCribing of doctors in Training) realist review. *BMJ Open.* 2015;5(10): e009059. 10.1136/bmjopen-2015-009059 26493460 PMC4620181

[39] Rycroft-Malone J McCormack B Hutchinson AM : Realist synthesis: illustrating the method for implementation research. *Implement Sci.* 2012;7(1): 33. 10.1186/1748-5908-7-33 22515663 PMC3514310

[40] Kantilal K Hardeman W Whiteside H : Realist review protocol for understanding the real-world barriers and enablers to practitioners implementing self-management support to people living with and beyond cancer. *BMJ Open.* 2020;10(9): e037636. 10.1136/bmjopen-2020-037636 32883731 PMC7473657

[41] Dada S Dalkin S Gilmore B : Applying and reporting relevance, richness and rigour in realist evidence appraisals: advancing key concepts in realist reviews. *Res Synth Methods.* 2023;14(3):504–514. 10.1002/jrsm.1630 36872619

[42] Page MJ McKenzie JE Bossuyt PM : The PRISMA 2020 statement: an updated guideline for reporting systematic reviews. *BMJ.* 2021;372:n71. 10.1136/bmj.n71 33782057 PMC8005924

[43] Weetman K Wong G Scott E : Improving best practice for patients receiving hospital discharge letters: a realist review. *BMJ Open.* 2019;9(6): e027588. 10.1136/bmjopen-2018-027588 31182447 PMC6561435

[44] Howe J MacPhee M Duddy C : A realist review of medication optimisation of community dwelling service users with serious mental illness. *BMJ Qual Saf.* 2025;34(1):40–52. 10.1136/bmjqs-2023-016615 38071586 PMC11671929

[45] Price T Brennan N Wong G : Remediation programmes for practising doctors to restore patient safety: the RESTORE realist review. *Health Serv Deliv Res.* 2021;9(11):1–116. 10.3310/hsdr09110 34043304

