# Supplementary material for: improving Pain mAnagement for childreN and young people attendeD by Ambulance (PANDA): protocol for a realist review
Source: NIHR Open Res. 2025 Jan 30;4:42. Originally published 2024 Jul 23. [Version 3] doi: 10.3310/nihropenres.13627.3 (PMC11443194; doi:10.3310/nihropenres.13627.3)
Supplement: Peer review history

# Reviewer report: Reviewer response for version 3

**DOI:** 10.3310/nihropenres.15093.r34668

**Published:** 2025-02-06

**Authors:** Simit Sapkota, Sunil Shrestha

**Affiliations:** 1 Kathmandu Cancer Center Hospital, Kathmandu, Central Development Region, Nepal; 2 Department of Research and Acdemics, Kathmandu Cancer Center, Kathmandu, Central Development Region, Nepal

The authors have answer all our queries. The article is approved without reservation.

Is the study design appropriate for the research question?

Yes

Is the rationale for, and objectives of, the study clearly described?

Yes

Are sufficient details of the methods provided to allow replication by others?

Partly

Are the datasets clearly presented in a useable and accessible format?

Not applicable

Reviewer Expertise:

I am a clinical oncologist working in Nepal, with a special interest in pain management and palliative care. I have been trying to formulate a rational use of opioids within the country.

We confirm that we have read this submission and believe that we have an appropriate level of expertise to confirm that it is of an acceptable scientific standard.

# Reviewer report: Reviewer response for version 3

**DOI:** 10.3310/nihropenres.15093.r34669

**Published:** 2025-01-31

**Authors:** Andrit Lourens

**Affiliations:** 1 University of Cape Town, Rondebosch, Western Cape, South Africa; 2 Health Sciences, Namibia University of Science and Technology, Windhoek, Khomas Region, Namibia; 3 Health Sciences, Higher Colleges of Technology, Sharjah, Sharjah, United Arab Emirates

No additional comments

Is the study design appropriate for the research question?

Yes

Is the rationale for, and objectives of, the study clearly described?

Yes

Are sufficient details of the methods provided to allow replication by others?

Yes

Are the datasets clearly presented in a useable and accessible format?

Not applicable

Reviewer Expertise:

Published several articles related to acute pain management in the prehospital setting as well as published evidence synthesis articles.

I confirm that I have read this submission and believe that I have an appropriate level of expertise to confirm that it is of an acceptable scientific standard.

# Reviewer report: Reviewer response for version 2

**DOI:** 10.3310/nihropenres.14914.r32872

**Published:** 2024-10-08

**Authors:** Simit Sapkota, Sunil Shrestha

**Affiliations:** 1 Kathmandu Cancer Center Hospital, Kathmandu, Central Development Region, Nepal; 2 Department of Research and Acdemics, Kathmandu Cancer Center, Kathmandu, Central Development Region, Nepal

The PANDA study protocol addresses an important issue in pediatric prehospital pain management but could benefit from refinements to enhance clarity and impact.

1. A realist approach is appropriate but insufficient detail on how stakeholder input will systematically shape the Initial Programme Theory (IPT). Greater clarity on the theoretical model for pain management would strengthen the conceptual foundation.
2. Including a wide range of sources is good, but more detail is needed on how relevance and rigor will be assessed, particularly for non-research documents. Justification for restricting to English-language and post-2000 documents should be included.
3. The tools for assessing pain in children are mentioned, but the limitations of these tools and how they may affect the synthesis need more discussion. The chosen threshold for pain relief (a reduction of 2 points on a 10-point scale) should also be better justified.
4. While the CMOC process is well-planned, the manuscript should specify how reviewer bias will be minimized during coding and synthesis. Handling contradictions in the literature also requires more explanation.
5. The protocol should extend its focus beyond developing a programme theory to outline potential interventions, ensuring practical relevance.
6. More clarity is needed on how input from PPI groups will be integrated into the review process and how differing views between stakeholders will be managed.
7. The process for assessing study rigor should be clarified with specific criteria, even if formal checklists aren't used. Additionally, themes like trauma-informed care that may emerge during synthesis should be given attention.

A few additional comments that could further strengthen the PANDA protocol:

1. While including PPI groups is commendable, the protocol could benefit from ensuring that stakeholders, especially ambulance clinicians, represent diverse geographic areas and levels of experience. Variations in regional policies, resource availability, and clinician experience can significantly impact pain management practices.
2. To maximize the utility of the findings, it would be beneficial to outline how the programme theory might be adapted or applied to international settings where ambulance systems and regulations differ from the UK. This would broaden the impact of the review and make it more globally relevant.
3. Given the complexity of pediatric pain management, including input from other medical disciplines (e.g., pediatricians, pain specialists) in addition to ambulance clinicians may provide a more holistic perspective, particularly on pharmacological and non-pharmacological interventions.
4. The focus of the review is primarily on acute pain management, but considering the long-term outcomes of prehospital pain experiences, such as the risk of developing chronic pain or PTSD, could add valuable insight into how early interventions may prevent later complications.

The PANDA protocol is well-structured and tackles a critical gap in prehospital pediatric care. Addressing the above points will improve its clarity and practical impact.

Is the study design appropriate for the research question?

Yes

Is the rationale for, and objectives of, the study clearly described?

Yes

Are sufficient details of the methods provided to allow replication by others?

Partly

Are the datasets clearly presented in a useable and accessible format?

Not applicable

Reviewer Expertise:

I am a clinical oncologist working in Nepal, with a special interest in pain management and palliative care. I have been trying to formulate a rational use of opioids within the country.

We confirm that we have read this submission and believe that we have an appropriate level of expertise to confirm that it is of an acceptable scientific standard, however we have significant reservations, as outlined above.

# Response

**Published:** 2025-01-27

**Authors:** Gregory Whitley

**Affiliations:** Clinical Audit and Research Un, East Midlands Ambulance Service NHS Trust, Lincoln, UK

Thank you for your time taken to review our manuscript. Your comments are very much appreciated. We have provided responses below:

- Greater detail on how the involved stakeholders shaped the initial programme theory will be detailed in the final review paper.
- We feel that the description of our assessment of relevance and rigour is adequate. These steps will be described further in the final review paper.
- We have amended some text to justify the language restriction placed on the review: “only documents reported in English (due to time and resource constraints)”
- We have amended some text to justify the date restriction placed on the review: “we will restrict our search to documents published from January 2000 onwards, due to known limited evidence prior to 2000 ^5^”
- We have added references to support the claim that a 2-point reduction in pain is clinically meaningful. In realist reviews, qualitative data plays a much greater role in synthesis than quantitative data, due to its explanatory value. We will acknowledge the limitations of pain assessment tools and their impact on the synthesis within the final review paper.
- We have explained that 10% of coding will be checked by a second reviewer, and we have added some text to explain how bias was reduced during synthesis. We have also explained in the protocol that we may juxtapose, reconcile, adjudicate, consolidate or situate findings – this will help to address contradictions in the literature.
- We certainly will not stop at the development of a realist programme theory. Under the Methods - Study Design section we explain that this review forms the first part of a sequence of studies that we hope will lead to the development and testing of a complex intervention. We have already added a sentence to the Programme theory refinement section to reiterate this and provide a sense of forward momentum at the end of the paper. This was to address comment #5 from peer-reviewer 2.
- The manuscript explains how patient and public involvement will be achieved within the review at the start of the methods section.  We have explained that both groups will be involved at the start and end of the review. We will elaborate on the exact nature of their involvement in the final review paper.
- We understand the request for more detail regarding the rigour assessment, however we feel we have provided enough detail. We specified the criteria in the Rigour section, which involved assessing the description of methods to generate data and the listing of references for evidence sources. We are unable to speculate on potential themes that may or may not arise from the synthesis process.
- We have commented on the diversity of the YPAG group. The ambulance clinician group was small on purpose to maximise participation during discussions. We ensured the selection of ambulance clinicians from difference areas of practice, including clinical practice, education and leadership. We have added a comment regarding the geography of the ACAG group.
- We will be including documents from the international landscape and in the final review paper we will comment on the scope of generalisability of the review findings. It would be beyond the scope of the protocol to comment on the scope of generalisability.
- We agree with your comments about including an interdisciplinary team in this review. The Working Group contained members with experience from the fields of General Practice, Nursing, Paramedicine and Psychology. A study Steering Committee, containing consultant pediatric emergency medicine physicians, also had oversight and input into this review – these members have been acknowledged.
- Thank you for the comment about long-term outcomes of prehospital pain experiences. We are anticipating this will come out in the review findings.

We hope you agree our manuscript is much improved as a result, and we hope you enjoy the final review paper when published.

# Reviewer report: Reviewer response for version 2

**DOI:** 10.3310/nihropenres.14914.r32837

**Published:** 2024-09-30

**Authors:** Andrit Lourens

**Affiliations:** 1 University of Cape Town, Rondebosch, Western Cape, South Africa; 2 Health Sciences, Namibia University of Science and Technology, Windhoek, Khomas Region, Namibia; 3 Health Sciences, Higher Colleges of Technology, Sharjah, Sharjah, United Arab Emirates

Thank you for considering my comments/suggestions and revising the protocol. I have no further additions to make.

Is the study design appropriate for the research question?

Yes

Is the rationale for, and objectives of, the study clearly described?

Yes

Are sufficient details of the methods provided to allow replication by others?

Yes

Are the datasets clearly presented in a useable and accessible format?

Not applicable

Reviewer Expertise:

Published several articles related to acute pain management in the prehospital setting as well as published evidence synthesis articles.

I confirm that I have read this submission and believe that I have an appropriate level of expertise to confirm that it is of an acceptable scientific standard.

# Reviewer report: Reviewer response for version 2

**DOI:** 10.3310/nihropenres.14914.r32836

**Published:** 2024-09-10

**Authors:** Heli Salmi

**Affiliations:** 1 Helsinki University, Helsinki, Finland

Thank you for the revised article! I have no further suggestions.

Is the study design appropriate for the research question?

Yes

Is the rationale for, and objectives of, the study clearly described?

Yes

Are sufficient details of the methods provided to allow replication by others?

Yes

Are the datasets clearly presented in a useable and accessible format?

Yes

Reviewer Expertise:

paediatric critical care

I confirm that I have read this submission and believe that I have an appropriate level of expertise to confirm that it is of an acceptable scientific standard.

# Reviewer report: Reviewer response for version 1

**DOI:** 10.3310/nihropenres.14796.r32432

**Published:** 2024-08-14

**Authors:** Heli Salmi

**Affiliations:** 1 Helsinki University, Helsinki, Finland

The authors have decided to conduct a study on how children in pain are currently managed by their prehospital emergency care providers. Then, according to literature and the current practices and circumstances, they will identify ideas for improvement, ands strategies for improvement. Ultimately, they will provide a framework about a programme.

 I am happy that the authors have decided to address such an important topic in paediatric critical care. I am not familiar with the research tools the authors describe, but they seem solid and relevant. I have a few suggestions for amendments to the protocol and to the article.

 I think this study protocol presentation would benefit from a “clinical touch”, both in order to make it more feasible and easier to understand for other clinicians. To meet this end,

- please explain the EMS system/ prehospital care in your context for a reader coming outside of the UK. Who attends to severely injured children in the prehospital setting? A nurse, a paramedic, a non-specialist doctor, an emergency physician, an anaesthesiologist, a paediatric anaesthesiologist? Or does it depend on the region or the case or other facts? Does this influence the choice of medications available? How are the care providers educated for paediatric pain and do all of them attend to children?
- Please discuss all relevant analgesic measures. If fentanyl is not available, what about other opioids? intravenously administered opioids? (es-)ketamine? what about adjuvant sedation and airway management to enable sufficient pain relief?

- please explain how pain is rated in your system. If the targeted pain relief outcome is at least -2 p on the scale, what scale (I presume it to be NRS or VAS or similar – but how is it assessed in infants and toddlers?)
- Perhaps the analysis should include a measure aiming at understanding how pain in children is evaluated in prehospital care. This may be the reason why children are undermedicated (if the personnel is only trained for pain score ratings, by definition all children under school age will not be able to provide a scale and are therefore at risk of not being medicated; also, the treatment response (-2 on the scale) will be impossible to achieve. Based on the results you will have, should your project also include a training tool for evaluating pain in children of different age?
- Your project is very important and ambitious. I congratulate you on that. When you’ll go as far, why leave it to a “framework for a programme” phase and not develop a full, even preliminary, programme for better pain relief for children in the prehospital care instead? I am afraid there is a risk that without this aim, the study will miss a final clinical aspect and remain too abstract to be easily used for education in the prehospital services. If, on the other hand, you wrote this with a final clinical programme in mind, your conclusions and suggestions for future would be more easily read and adopted to practice by the busy clinicians in the field. To my understanding, the study setting would enable this kind of development.

Is the study design appropriate for the research question?

Yes

Is the rationale for, and objectives of, the study clearly described?

Yes

Are sufficient details of the methods provided to allow replication by others?

Yes

Are the datasets clearly presented in a useable and accessible format?

Yes

Reviewer Expertise:

paediatric critical care

I confirm that I have read this submission and believe that I have an appropriate level of expertise to confirm that it is of an acceptable scientific standard, however I have significant reservations, as outlined above.

# Response

**Published:** 2024-09-02

**Authors:** Gregory Whitley

**Affiliations:** Clinical Audit and Research Un, East Midlands Ambulance Service NHS Trust, Lincoln, UK

Thank you for your thorough review of our manuscript. We appreciate your comments about needing a more clinical focus and have responded accordingly. We now feel that the manuscript is much improved. We have addressed the following points:

1. We have added a section to the introduction to give local context to the EMS system in the UK. We have described the scope of analgesic availability across the different clinician levels and described typical pain assessment processes. We have already alluded to the limited education and training around pain management for CYP in the first paragraph and so did not wish to labour this point any further.
2. We agree that pain assessment should form a core component of the synthesis. We will perform synthesis on all included documents and where able, we will develop CMOCs related to pain assessment.  It is likely that the programme theory resulting from this review will allude to the pain assessment process, however we are unable to speculate at this stage. We also agree that a training tool to facilitate the pain assessment process in CYP would be helpful. Again, we anticipate this coming out in the synthesis of this review somewhat, and in the subsequent stages of the PANDA Study, and it is likely to form a component of the future complex intervention, however we are unable to speculate on this and wish to be led by the data.
3. We appreciate your kind comments and agree this is important and ambitious work. We certainly will not stop at the development of a realist programme theory. Under the Methods - Study Design section we explain that this review forms the first part of a sequence of studies that we hope will lead to the development and testing of a complex intervention. We have added a sentence to the Programme theory refinement section to reiterate this and provide a sense of forward momentum at the end of the paper.

# Reviewer report: Reviewer response for version 1

**DOI:** 10.3310/nihropenres.14796.r32435

**Published:** 2024-08-13

**Authors:** Andrit Lourens

**Affiliations:** 1 University of Cape Town, Rondebosch, Western Cape, South Africa; 2 Health Sciences, Namibia University of Science and Technology, Windhoek, Khomas Region, Namibia; 3 Health Sciences, Higher Colleges of Technology, Sharjah, Sharjah, United Arab Emirates

**Peer-review Report:**

 Thank you for the opportunity to peer-review the realist review protocol titled ‘improving Pain mAnagement for childreN and young people attendeD by Ambulance (PANDA): protocol for a realist review.’ aiming to understand how ambulance clinicians can provide improved prehospital acute pain management for Children and Young People. The protocol proposes a five-step approach for conducting the review following the RAMESES guidance to create context-mechanism-outcome configurations and test and refine the Initial Programme Theory into a realist Programme Theory.

 Overall, the protocol is well-written and structured with the methods describing the core steps required to ensure scientific soundness and reproducibility. I am confident that this review will contribute positively to the field of study, and I wish all involved the best with the overall research project. I do, however, have some comments and suggestions detailed below.

**Comments:**

**Abstract:**

***Methods:***

 As per my comment below ‘perform screening in duplicate’ is captured under step 2 of the methods of the study, however, screening is part of the selection of the sources to be included which is step 3. I would suggest that the authors move it to step 3.

**Introduction:**

 Overall, the introduction is well-written and concise providing general background information, what is known and not known about the topic in the specific population and articulating the rationale for conducting the realist review. Two minor comments for the authors to consider.

 Please consider reviewing the last sentence of the first paragraph of the introduction. The sentence is quite long, and it is challenging to read and understand the first time. I would suggest ending the sentence after ‘… variety of barriers’ followed by discussing the barriers in a subsequent sentence or two. My comment relates more to readability and understanding of the contents of the sentence, the information provided is valid and relevant.

 After abbreviating the term ‘children and young people (CYP)’, I would suggest that the abbreviation is used consistently throughout the introduction and the rest of the manuscript. I would also suggest that the authors ensure that all abbreviations are used consistently throughout the protocol manuscript once abbreviated. There are some inconsistencies in the manuscript.

**Methods:**

***Patient and Public Involvement***

 In the first paragraph of the section, the abbreviation ‘IPT’ is used, however, the term is only abbreviated later in the methods section. In addition, the term is abbreviated a few subsequent times. Please consider correcting this.

 In the first sentence of the second paragraph of the section, the word ‘group’ appears twice *‘* *(the Healthier Ageing Patient and Public Involvement group (HAPPI) Group))*. Perhaps the first ‘group’ is unnecessary.

***Step 2: Searching and Screening***

 I would suggest that ‘The Cochrane Library’ be moved to the list of bibliographic databases searched instead of grey literature. Although ‘The Cochrane Library’ can provide access to a broad array of source documents including protocols of ongoing trials/systematic reviews, it is predominantly a source of completed systematic reviews and RCTs. The sub-heading ‘Grey Literature Search’ could be changed to ‘Searching Other Resources’ as a more commonly used sub-heading allowing for broader inclusion.

***Inclusion Criteria***

 For the population inclusion criteria please change the bullet point “Ambulance clinicians (including but not limited to paramedics, emergency medical technicians, prehospital emergency nurses) who attend **to CYP** suffering acute pain.”

 Documents published from January 2000 onwards should rather be a search restriction and documents reported in English, an inclusion criterion. For both, the authors should consider provide a justification for the decision to exclude publications before 2000 and non-English documents.

***Exclusion Criteria***

 I would argue that the last paragraph under this section discussing the screening of the search results against the eligibility (inclusion and exclusion) criteria is part of ‘Step 3: Document Selection’ as it is part of the stages of selecting sources for the review. Please move to Step 3. In line with this, the headings for Step 2 should be revised. Suggested options for heading ‘Step 2: Evidence Search’

***Step 3: Document Selection***

 In evidence synthesis, the results of searches and the screening process are commonly reported using a PRISMA flow diagram. Please consider adding that the selection of included sources will be reported using a PRISMA flow diagram. The reference for a PRISMA flow diagram is already in the reference list (reference 39).

 For the screening of sources and the assessment of relevance and rigour, please indicate whether the duplicate screening/assessment will be conducted independently.

**Rigour**

 Please check the spelling of rigour in the second sentence under the sub-heading ‘Rigour’.

**Step 4: Extracting and organising data**

 Although the authors describe that coding will be done by one reviewer and 10% checked by a second reviewer, this detail is not described for the data extraction process. Please describe this (how many reviewers will be involved, how will the reviewer(s) extract the data, and how will the data be checked) for the data extraction process as well.

**Programme theory refinement**

 Please review the sentence to read ‘The programme theory developed from a recent realist review will be used **as** a framework to develop our realist programme theory.

Is the study design appropriate for the research question?

Yes

Is the rationale for, and objectives of, the study clearly described?

Yes

Are sufficient details of the methods provided to allow replication by others?

Yes

Are the datasets clearly presented in a useable and accessible format?

Not applicable

Reviewer Expertise:

Published several articles related to acute pain management in the prehospital setting as well as published evidence synthesis articles.

I confirm that I have read this submission and believe that I have an appropriate level of expertise to confirm that it is of an acceptable scientific standard, however I have significant reservations, as outlined above.

# Response

**Published:** 2024-09-02

**Authors:** Gregory Whitley

**Affiliations:** Clinical Audit and Research Un, East Midlands Ambulance Service NHS Trust, Lincoln, UK

Thank you for your thorough review of our manuscript. We appreciate your comments and have responded accordingly. We now feel that the manuscript is much improved. We have addressed the following points:

1. We are unable to move the statement re. screening in duplicate to Step 3 because Step 2 and 3 are distinct. We screened documents in duplicate for Step 2, and then assessed relevance and rigour in duplicate for Step 3. The assessment of relevance did inform inclusion, whereas the assessment of rigour did not. We feel it is important to make the statements re. duplicate screening and duplicate assessment for Step 2 and Step 3 separately.  We have also relabelled Step 3 for further clarity.
2. We have edited the sentence in the Introduction to improve readability.
3. We have replaced all references to “children and young people” with the abbreviation “CYP” after its first definition.
4. We have addressed the error re. the definition of “IPT”.
5. We have addressed the error re. the definition of “HAPPI” group.
6. We have moved The Cochrane Library to the Database Search and rephrased “grey literature search” to “searching other resources”.
7. We have edited the inclusion criteria as suggested and provided a justification for searching from January 2000 onwards.
8. We understand the confusion and similarity between Step 2 Searching and *screening*, and Step 3 *Document selection*. According to realist methodology these are distinct steps. We have renamed Step 3 to “Relevance and rigour assessment” for clarity.
9. We have stated that number of included documents will be reported using a PRISMA flow diagram and clarified that all duplicate screening and assessment will be conducted separately.
10. We have amended the spelling of “rigor”.
11. We have clarified that data extract of document characteristics will be performed by one reviewer and verified by a second. We will not extract data for the purpose of analysis per se, but rather we will upload the full documents for initial coding – we have clarified this.
12. We have edited the sentence regarding programme theory refinement.
